# The value of HDL subfractions in predicting cardiovascular outcomes in untreated, diabetic patients with stable coronary artery disease: An age- and gender-matched case-control study

**DOI:** 10.3389/fendo.2022.1041555

**Published:** 2023-01-12

**Authors:** Wei Zhang, Jing-Lu Jin, Hui-Wen Zhang, Ya-Xin Zhu, Qian Dong, Jing Sun, Yuan-Lin Guo, Ke-Fei Dou, Rui-Xia Xu, Jian-Jun Li

**Affiliations:** State Key Laboratory of Cardiovascular Disease, Cardiometabolic Medicine Center, Fuwai Hospital, Chinese Academy of Medical Sciences & Peking Union Medical College, Beijing, China

**Keywords:** cardiovascular events, coronary artery disease, diabetes, high-density lipoprotein subfractions, predicting value

## Abstract

**Objective:**

The aim of the present study was to examine the value of high-density lipoprotein (HDL) subfractions for predicting cardiovascular events (CVEs) in untreated type 2 diabetes mellitus (T2DM) patients with stable coronary artery disease (SCAD) using an age- and gender-matched case-control study.

**Methods:**

In total, 185 SCAD patients and 185 T2DM patients with SCAD were enrolled and subjected to a clinical follow-up of CVEs. HDL subfractions were analyzed using the Quantimetrix Lipoprint System. The relationship between HDL subfractions and CVEs in T2DM patients with SCAD was evaluated by Kaplan–Meier analysis and Cox proportional hazard models.

**Results:**

During a median 37.7-month follow-up, T2DM patients with SCAD had a higher percentage of CVEs compared to SCAD patients (p=0.039). The concentration of the combined intermediate and small HDL-C subfraction (defined as the mixed HDL subfraction) was related to the event incidence in T2DM patients with SCAD (p=0.004), and it was positively associated with increased CVEs even after adjustment in three models. Kaplan-Meier curve analysis indicated that T2DM patients with SCAD in the high mixed HDL subfraction group (>28 mg/dL) had lower event-free survival rates (p=0.008).

**Conclusions:**

Elevated concentration of the mixed HDL subfraction predicts events in T2DM patients with SCAD.

## Introduction

Cardiovascular disease (CVD) is one of major causes of death in the world with atherosclerotic cardiovascular disease (ASCVD) accounting for half of these deaths. Patients with type 2 diabetes mellitus (T2DM) are at a 2- to 4-fold increased risk of ASCVD, which accounts for the leading cause of mortality (1). Dyslipidemia in T2DM is characterized by hypertriglyceridemia, normal to mildly elevated levels of low-density lipoprotein-cholesterol (LDL-C; with increased levels of small dense LDL particles) and low high-density lipoprotein-cholesterol (HDL-C) levels (with variable changes in HDL composition) (2). Previous study has indicated that atherosclerotic lesions of the vascular system in T2DM patients is not only dependent on glycemic control but also the presence of coexisting risk factors, including dyslipidemia (3).

HDL is highly heterogenous in its size and composition, and HDL subclasses are generally classified as HDL2 (larger and less dense) and HDL3 (smaller and denser) (4). The conformational and functional properties of HDL particles may be altered by several factors. The findings of lipid-lowering drugs that increase HDL-C but do not reduce cardiovascular events (CVEs) or atherosclerosis have attracted an interest in alternative indexes of HDL quantity [i.e., HDL particle or apolipoprotein A1 (ApoA1)] or HDL quality, such as particle size, subclass distribution or various measures of HDL functionality (5, 6). Moreover, a previous study has shown that the transformation of HDL particles into pathogenic ones occurs in T2DM (7). Garvey et al. (8) demonstrated that lipoprotein subclass alterations in T2DM are moderately exacerbated as evidenced by a decrease in HDL size as a result of depletion of large HDL particles and a modest increase in small HDL. Furthermore, our group previously identified significant associations between HDL-C subfractions and T2DM or stable coronary artery disease (SCAD) (9). Thus, it is important to understand these features to help improve the prevention of macrovascular complications.

Although previous studies have shown the prognostic role of dyslipidemia in T2DM patients with SCAD, the effects of plasma HDL-C subfractions on cardiovascular outcomes in T2DM patients with SCAD are unclear. Thus, the aim of the present study was to investigate the relationship between HDL-C subfractions and the risk of CVEs in SCAD patients or T2DM patients with SCAD.

## Materials and methods

### Study design and population

**Figure 1:**
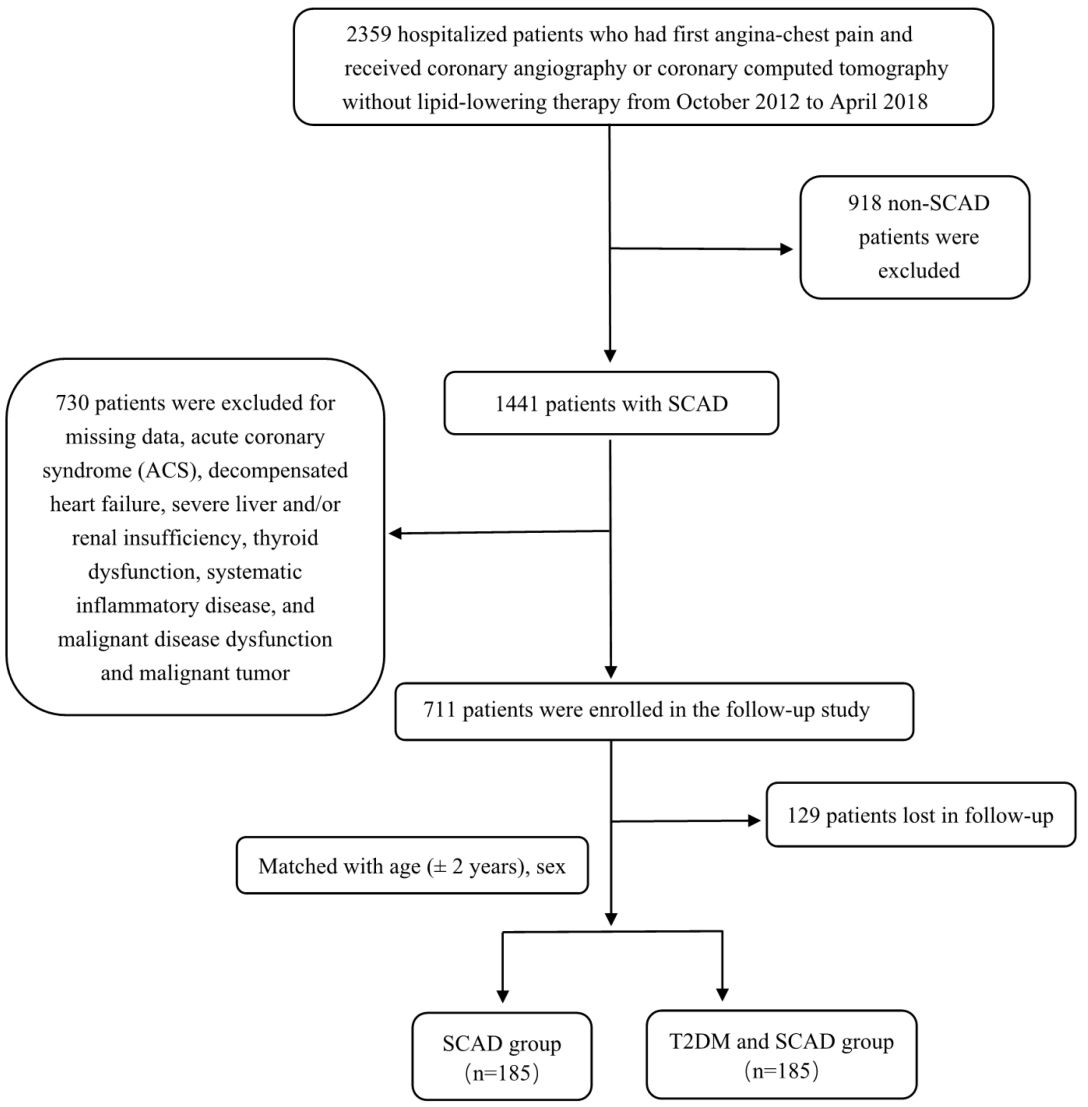
Flowcharts of patient selection and grouping.

A total of 2359 participants with angina-like chest pain and/or positive treadmill exercise test or clinically suspected CVD from Fuwai Hospital were consecutively enrolled between October 2012 and April 2018. The exclusion criteria of patients were as follows: 1) aged <18 years old; 2) treatment history of lipid-lowering drugs and antidiabetic drugs prior to entering the study; 3) severe end-stage diseases, such as renal and/or liver dysfunction, heart failure and malignant carcinoma; 4) systematic inflammatory disease or severe infection; 5) thyroid disorder; and 6) pregnancy. In total, 918 patients were excluded due to the lack of angiography-proven SCAD. SCAD was defined as typical angina-like chest pain brought on by exertion and relieved by rest or sublingual nitrates or both, a positive treadmill exercise test >1-mm ST-segment depression) and stable obstructive lesion >50% in at least 1 of the 3 major coronary arteries or major branches assessed by at least 2 independent senior interventional cardiologists. Another 730 patients were excluded due to the following reasons: acute coronary syndrome (ACS); decompensated heart failure**;** severe liver and/or renal insufficiency**;** thyroid dysfunction**;** systematic inflammatory disease; malignant disease dysfunction; and malignant tumor. All patients in the present study were followed up every year by clinic revisit or by phone interview with trained nurses or doctors blinded to the clinical data until the first CVE occurred or up to the last day of the follow-up period. In total, 711 patients were enrolled for follow-up, but 129 patients were lost to follow-up, resulting in 582 patients with intact follow-up information. Among these patients, there were 185 T2DM patients with SCAD according to the definition. From the remaining pool of patients (n=397), we randomly selected control subjects at a 1:1 ratio matched with the same sex, and the age difference was ± 2 years old. Thus, there were 185 T2DM patients with SCAD and 185 SCAD patients in the final analysis. CVEs were defined as follows: hospitalized unstable angina; non-fatal myocardial infarction (MI); stroke; unplanned percutaneous coronary intervention or coronary artery bypass grafting; and cardiovascular death. Non-fatal MI was diagnosed as positive cardiac troponins along with typical chest pain or typical electrocardiogram serial changes. Cardiovascular death was diagnosed based on death primarily caused by acute myocardial infarction (AMI), congestive heart failure, malignant arrhythmia and other structural or functional cardiac diseases. Follow-up time was calculated as the number of months from the enrollment till the last traceable hospital outpatient record, hospital inpatient record or telephone interview before April 2019. A study population enrollment flowchart is presented in **Figure 1**.

T2DM was diagnosed by fasting plasma glucose (FPG) 7.0 ≥ mmol/L, 2-h plasma glucose of oral glucose tolerance test ≥ 11.1 mmol/L, hemoglobin A1c (HbA1c) level ≥ 6.5%, currently using antidiabetic drugs or currently using insulin. Hypertension was diagnosed as medical history of hypertension, currently receiving antihypertensive drugs or hospital recorded systolic blood pressure (SBP) ≥ 140 mmHg and/or diastolic blood pressure (DBP) ≥ 90 mmHg for three or more consecutive times. The body mass index (BMI) was calculated as weight (kg) divided by height (m) squared. Patients who had a smoking habit of at least one cigarette per day on admission were classified as current smokers. Patients who reported alcohol intake at least once a week were classified as a current drinker. Family history of CAD was defined when myocardial ischemia or infarction was documented in at least one first-degree relative. Baseline medications (medications before admission) were collected by interviewing or from hospital-recorded medical history. Patients who received statin therapy at the time of discharging from hospital were also recorded.

The present study fully complied with the Declaration of Helsinki and received approval from the Ethics Committee of Fu Wai Hospital and Cardiovascular Institute (Beijing, China). All included patients provided prior written consent.

### Blood sample measurement

Blood samples were collected into EDTA-containing tube**s**. After centrifugation at 3000 rpm for 10 min at 4°C, plasma was collected and stored at −80°C. Plasma concentrations of total cholesterol (TC), triglyceride (TG), LDL-C and HDL-C were measured by an automatic biochemistry analyzer (Hitachi 7150, Tokyo, Japan) using an enzymatic assay. HbA1c was measured using the Tosoh Automated Glycohemoglobin Analyzer (HLC-723G8, Tokyo, Japan). Plasma concentrations of ApoA1, apolipoprotein B (ApoB) and lipoprotein (a) [Lp(a)] were measured using a turbidimetric immunoassay with the automatic biochemistry analyzer mentioned above. Lipoprotein subfraction analysis was performed using the Lipoprint System (Quantimetrix Corporation, Redondo Beach, CA, USA) according to the manufacturer’s instructions. The HDLs were divided into 10 subfractions as follows: subfractions 1–3 indicated large HDL particles; subfractions 4–7 represented intermediate HDL particles; and subfractions 8–10 represented small HDL particles. Subsequently, the cholesterol concentration (mg/dL) and lipoprotein subfraction proportion (%) were determined for each lipoprotein subfraction.

### Statistical analysis

All statistical analyses were performed using statistical package for social science (SPSS) version 25 (IBM Corporation, Armonk, New York, USA). Continuous variables with normal distribution are presented as mean ± standard deviation (SD), and variables with abnormal distribution are expressed as the median (interquartile range). Categorical variables are presented as a number (frequency). The independent sample Student’s t-test or Mann–Whitney U test was performed to analyze the differences in variables among groups. χ2 analysis and Fisher’s test were performed to determine the statistical difference in categorical variables between two groups. The logistic regression analysis was performed to determine the relationship between HDL subfractions and T2DM prevalence. The hazard ratios (HRs) and 95% confidence intervals (CIs) were calculated by univariate and multivariate Cox regression analyses. The cumulative event-free survival rates of the high mixed HDL subfractions (>28 mg/dL) and the low mixed HDL subfractions (≤ 28 mg/dL) were estimated by the Kaplan–Meier curve with the log-rank test. Moreover, p<0.05 was considered statistically significant.

## Results

### Baseline characteristics

The demographics and clinical characteristics of T2DM patients with SCAD and SCAD patients are summarized in **Table 1**. The average age was 59.08 ± 9.67 years, and males accounted for 70.3% (n = 260) of the enrolled patients. Compared to the SCAD group, the T2DM and SCAD group had significantly higher levels of glucose, HbA1C, TG, TC, LDL-C, ApoB, free fatty acids (FFAs) and BMI (p<0.001, p<0.001, p<0.001, p=0.002, p=0.014, p<0.001, p=0.001 and p=0.001, respectively). Moreover, the T2DM and SCAD group had a higher morbidity of hypertension and dyslipidemia compared to the SCAD group. The levels of HDL-C and Lp(a) in the T2DM and SCAD group displayed a descending tendency, and the ApoA1 level showed an increasing tendency; however, the differences were not statistically significant (p=0.283, p=0.422 and p=0.118, respectively). Moreover, the T2DM and SCAD group had a higher concentration and percentage of the small HDL-C subfraction (p=0.002 and p<0.001, respectively) compared to the SCAD group. Nevertheless, the concentration and percentage of the large HDL-C subfraction was significantly reduced in the T2DM and SCAD group (p=0.001 and p<0.001, respectively) compared to the SCAD group. In addition, no significant difference was observed in the concentration and percentage of the intermediate HDL-C subfraction between the two groups.

**Table 1:** Baseline characteristics in this study.

Variable	Total	SCAD	T2DM and SCAD	p-value
	(n=370)	(n=185)	(n=185)	
Clinical Characteristics				
Age (years)	59.08 ± 9.67	59.43 ± 9.87	58.73 ± 9.48	0.488
Men [n (%)]	260 (70.3)	130 (70.3)	130 (70.3)	0.845
BMI (kg/m^2^)	25.93 ± 3.43	25.37 ± 3.14	26.51 ± 3.61	**0.001**
Hypertension [n (%)]	249 (67.3)	114 (61.6)	135 (73.0)	**0.016**
Smoking [n (%)]	143 (38.6)	65 (35.1)	78 (42.2)	0.359
Alcohol consumption [n (%)]	84(22.8)	47(25.4)	37(20.0)	0.478
FH of CAD [n (%)]	69(18.6)	38(20.5)	31 (16.8)	0.363
Dyslipidemia [n (%)]	239 (64.6)	106 (57.3)	133 (71.9)	**0.003**
Laboratory parameters				
Glucose (mmol/L)	5.04 ± 0.58	5.04 ± 0.58	6.88 ± 1.88	**<0.001**
HbA1C (%)	5.74 ± 0.37	5.74 ± 0.37	7.36 ± 1.27	**<0.001**
ESR (mm/h)	10.64 ± 11.36	9.85 ± 11.67	11.43 ± 11.00	0.182
ALT (U/L)	24.81 ± 23.02	24.93 ± 28.12	24.68 ± 16.46	0.919
AST (U/L)	19.77 ± 11.37	19.99 ± 12.59	19.54 ± 10.01	0.707
ALP (IU/L)	64.99 ± 17.83	64.58 ± 17.82	65.40 ± 17.89	0.660
GGT (IU/L)	37.51 ± 31.16	34.96 ± 27.84	40.07 ± 34.05	0.115
Cr (μmol/L)	74.98 ± 16.47	75.31 ± 15.38	74.64 ± 17.54	0.696
BUN (mmol/L)	5.85 ± 1.49	5.71 ± 1.35	6.00 ± 1.61	0.067
UA (mmol/L)	359.74 ± 91.55	365.11 ± 87.41	354.33 ± 95.47	0.295
Lipid Profile				
TG (mg/dL)	146.01(108.41-207.52)	130.09(99.56-185.73)	162.84(120.58-228.10)	**<0.001**
TC (mg/dL)	188.12 ± 37.81	182.01 ± 32.96	194.27 ± 41.31	**0.002**
HDL-C (mg/dL)	40.69 ± 10.58	41.29 ± 11.03	40.10 ± 10.11	0.283
LDL-C (mg/dL)	125.79 ± 34.20	121.45 ± 30.76	130.16 ± 36.91	**0.014**
ApoA1 (g/L)	1.31 ± 0.30	1.28 ± 0.31	1.33 ± 0.28	0.118
ApoB (g/L)	1.05 ± 0.26	0.99 ± 0.23	1.10 ± 0.27	**<0.001**
Lp (a) (mg/L)	134.40 (60.72-286.20)	143.97 (57.30-302.15)	126.28(65.80-267.37)	0.422
FFA (mmol/L)	0.45 ± 0.18	0.42 ± 0.16	0.49 ± 0.19	**0.001**
HDL subfractions				
Large HDL-C (mg/dL)	12.42 ± 5.68	13.37 ± 6.09	11.47 ± 5.07	**0.001**
Intermediate HDL-C (mg/dL)	20.04 ± 5.14	20.08 ± 5.71	20.00 ± 5.36	0.880
Small HDL-C (mg/dL)	8.43 ± 3.03	7.94 ± 2.74	8.92 ± 3.22	**0.002**
Large HDL-C (%)	29.59 ± 7.75	31.45 ± 7.57	27.72 ± 7.50	**<0.001**
Intermediate HDL-C (%)	49.30 ± 4.52	49.00± 4.64	49.61 ± 4.38	0.194
Small HDL-C (%)	20.94 ± 6.30	19.55 ± 5.80	22.34 ± 6.49	**<0.001**
Medications				
Baseline Aspirin	146(39.5)	74(40.0)	72(38.9)	0.864
Follow-up Aspirin	321(86.8)	157(84.9)	164(88.6)	0.343
Baseline CCB	91(24.6)	45(23.4)	46(25)	0.880
Follow-up CCB	300(81.1)	148(80.0)	152(82.1)	0.520
Baseline ACEI/ARB	65(17.6)	30(16.2)	35(18.9)	0.988
Follow-up ACEI/ARB	297(78.6)	142(76.8)	149(82.7)	0.321
Baseline β-blockers	73(19.7)	36(19.5)	37(20.0)	0.876
Follow-up β-blockers	151(40.8)	75(40.5)	76(41.1)	0.950
Follow up Statin	361(97.6)	180(97.3)	181(97.8)	0.479
Follow up antidiabetes drugs				
OADs	180(48.6)	–	180(97.3)	
Insulin	178(48.1)	–	178(96.2)	
**Cardiac Events (%)**	38(10.3)	13(7.0)	25(13.6)	**0.039**

Data were expressed as mean ± SD, median with 25th and 75th percentile, n (%).

ACEI/ARB, angiotensin-converting enzyme inhibitors/angiotensin receptor blocker; ALT, alanine aminotransferase; ALP, alkaline phosphatase; ApoA1, apolipoprotein A1; ApoB, apolipoprotein B; AST, aspartate aminotransferase; BMI, body mass index; BUN, blood urea nitrogen; CCB, calcium channel blocker; Cr, creatinine; ESR, erythrocyte sedimentation rate; FFA, free fatty acids; FH, family history; GGT, glutamyl transferase; HbA1C, hemoglobin A1C; HDL-C, high-density lipoprotein cholesterol; LDL-C, low-density lipoprotein cholesterol; Lp(a), lipoprotein (a); OADs, oral antidiabetes drugs; SCAD: stable coronary artery disease;SD: standard deviation; T2DM:type 2 diabetes mellitus;TC, total cholesterol; TG, triglyceride; UA, uric acid.

p < 0.05 suggests significant difference. The meaning of the bold values were only for indicating the corresponding indicators have significant difference (p < 0.05).

### Association of HDL subfractions in T2DM patients with SCAD

The median follow-up time was 37.7 months (interquartile range: 13.2–47.1 months). During the follow-up period, 13 (7.0%) patients in the SCAD group developed CVEs, and 25 (13.6%) patients in the T2DM and SCAD group developed CVEs; there was a significant difference between two groups (p=0.039). A logistic regression analysis was performed to further assess the association of HDL subfractions with the coexistence of T2DM and SCAD (**Table 2**). After adjusted logistic regression analysis, the concentration and percentage of the small HDL subfraction were significantly positively associated with the coexistence of T2DM and SCAD [unadjusted OR (95% CI): 1.122 (1.042–1.207), p=0.002; adjusted OR (95% CI): 1.128 (1.044–1.218), p=0.002; unadjusted OR (95% CI): 1.077 (1.040–1.115), p<0.001; adjusted OR (95% CI): 1.071 (1.032–1.112), p<0.001; respectively], while the concentration and percentage of the large HDL-C subfraction were inversely related to the coexistence of T2DM and SCAD [unadjusted OR (95% CI): 0.939 (0.903–0.977), p=0.002; adjusted OR (95% CI): 0.949 (0.909–0.992), p=0.019; unadjusted OR (95% CI): 0.936 (0.910–0.964), p<0.001; adjusted OR (95% CI): 0.939 (0.910–0.970), p<0.001; respectively].

**Table 2:** Associations of HDL subfractions with T2DM and SCAD by logistic regression analysis.

Variable	Unadjusted logistics regression analysis				Adjusted logistics regression analysis	
	OR	95% CI	p-value	OR	95% CI	p-value
HDL-C						
Large HDL-C(mg/dL)	0.939	0.903-0.977	**0.002**	0.949	0.909-0.992	**0.019**
Intermediate HDL-C(mg/dL)	0.997	0.958-1.037	0.879			
Small HDL-C(mg/dL)	1.122	1.042-1.207	**0.002**	1.128	1.044-1.218	**0.002**
Large HDL-C (%)	0.936	0.910-0.964	**<0.001**	0.939	0.910-0.970	**<0.001**
Intermediate HDL-C (%)	1.031	0.985-1.079	0.195			
Small HDL-C (%)	1.077	1.040-1.115	**<0.001**	1.071	1.032-1.112	**<0.001**

The adjusting known confounders were including age, gender, hypertension, family history of CAD, current smoking, BMI, drinking.

BMI, body mass index; CAD: coronary artery disease; CI, confidence intervals; HDL-C, high-density lipoprotein cholesterol; OR, odds Ratio; SCAD, stable coronary artery disease;T2DM, type 2 diabetes mellitus.

p < 0.05 suggests significant difference. The meaning of the bold values were only for indicating the corresponding indicators have significant difference (p < 0.05).

### Clinical and biochemical characteristics of patients in the T2DM and SCAD group with or without CVEs

The demographics and clinical characteristics of patients in the T2DM and SCAD group with or without CVEs are shown in **Table 3**. Patients with CVEs had significantly higher levels of ALT, AST and glutamyl transferase (GGT) (p=0.007, p=0.015 and p=0.033, respectively), while the ages of patients with CVEs were lower (p=0.038). Furthermore, patients with CVEs had higher intermediate HDL-C concentrations, small HDL-C concentrations and combined intermediate HDL-C and small HDL-C (defined as mixed HDL subfraction) concentrations (p<0.001, p=0.028 and p<0.001, respectively).

**Table 3:** Baseline characteristics in T2DM and SCAD with or without CVEs.

Variable	Total	Without CVEs	CVEs	p-value
	(n=185)	(n=160)	(n=25)	
Clinical Characteristics				
Age (years)	58.73 ± 9.48	59.62 ± 8.89	56.36 ± 10.63	**0.038**
Men [n (%)]	130 (70.3)	112 (70.0)	19 (76.0)	0.568
BMI (kg/m^2^)	26.51 ± 3.61	26.46 ± 3.38	26.78 ± 4.93	0.680
Hypertension [n (%)]	135 (73.0)	115 (71.9)	20 (80.0)	0.420
Smoking [n (%)]	78 (42.2)	66 (41.3)	12 (48.0)	0.812
Alcohol consumption [n (%)]	37(20.0)	30(18.8)	7(28.0)	0.543
FH of CAD [n (%)]	31(16.8)	25(15.6)	6(24.0)	0.304
Dyslipidemia [n (%)]	133 (71.9)	115 (71.9)	18 (72.0)	0.973
Laboratory parameters				
Glucose (mmol/L)	6.88 ± 1.88	6.88 ± 1.80	6.89 ± 2.37	0.982
HbA_1C_ (%)	7.36 ± 1.27	7.34 ± 1.28	7.42 ± 1.26	0.707
ESR (mm/h)	11.43 ± 11.00	10.80 ± 10.32	13.14 ± 12.61	0.200
ALT (U/L)	24.68 ± 16.46	22.69 ± 15.31	30.02 ± 18.32	**0.007**
AST (U/L)	19.54 ± 10.01	18.20 ± 8.32	23.14 ± 12.98	**0.015**
ALP (IU/L)	65.40 ± 17.89	65.18 ± 17.73	66.76 ± 19.20	0.683
GGT (IU/L)	40.07 ± 34.05	37.87 ± 31.89	54.08 ± 43.72	**0.033**
Cr (μmol/L)	74.64 ± 17.54	73.77 ± 16.38	76.97 ± 20.33	0.271
BUN (mmol/L)	6.00 ± 1.61	6.02 ± 1.60	5.94 ± 1.64	0.777
UA (mmol/L)	354.33 ± 95.47	347.20 ± 89.72	373.44 ± 108.07	0.130
Lipid Profile				
TG (mg/dL)	162.84 (120.58-228.10)	160.18 (113.05-216.37)	163.72(125.89-244.03)	0.310
TC (mg/dL)	194.27 ± 41.31	194.06 ± 41.84	195.58 ± 38.58	0.864
HDL-C (mg/dL)	40.10 ± 10.11	39.37 ± 10.02	42.05 ± 10.19	0.111
LDL-C (mg/dL)	130.16 ± 36.91	129.72 ± 36.67	131.34 ± 37.87	0.791
ApoA1 (g/L)	1.33 ± 0.28	1.32 ± 0.28	1.36 ± 0.28	0.363
ApoB (g/L)	1.10 ± 0.27	1.10 ± 0.26	1.11 ± 0.30	0.747
Lp (a) (mg/L)	126.28 (65.80-267.37)	130.10 (66.82-278.70)	98.46 (56.12-228.52)	0.386
FFA (mmol/L)	0.49 ± 0.19	0.48 ± 0.19	0.51 ± 0.18	0.343
HDL subfractions				
Large HDL-C (mg/dL)	11.47 ± 5.07	11.43 ± 5.24	11.60 ± 4.65	0.836
Intermediate HDL-C (mg/dL)	20.00 ± 5.36	19.41 ± 4.52	23.72 ± 8.21	**<0.001**
Small HDL-C (mg/dL)	8.92 ± 3.22	8.72 ± 2.91	10.24 ± 4.63	**0.028**
Mixed HDL (mg/dL)	27.72 ± 7.50	28.13 ± 6.38	33.96 ± 11.97	**<0.001**
Large HDL-C (%)	27.72 ± 7.50	27.80 ± 7.58	27.23 ± 7.11	0.727
Intermediate HDL-C (%)	49.61 ± 4.38	49.66 ± 3.97	49.26 ± 6.52	0.675
Small HDL-C (%)	22.34 ± 6.49	22.50 ± 6.54	21.36 ± 6.20	0.416
Mixed HDL (%)	71.95 ± 7.87	71.75 ± 7.85	72.47 ± 8.00	0.746
Medications				
Baseline Aspirin	72(38.9)	61(38.1)	11(44.0)	0.591
Follow-up Aspirin	66(35.7)	57(35.6)	9(36.0)	0.988
Baseline CCB	46(24.9)	43(26.9)	3(12.0)	0.106
Follow-up CCB	34(18.4)	32(20.0)	2(8.0)	0.146
Baseline ACEI/ARB	35(18.9)	29(18.1)	6(24.0)	0.495
Follow-up ACEI/ARB	24(13.0)	20(12.5)	4(16.0)	0.637
Baseline β-blockers	37(20.1)	32(20.1)	5(20.0)	0.988
Follow-up β-blockers	29(15.7)	25(15.6)	4(16.0)	0.972
Follow up Statin	76(41.1)	65(40.6)	11(44)	0.768
Follow up antidiabetes drugs				
OADs	92(49.7)	80(50.0)	12(48.0)	0.830
Insulin	48(25.9)	42(26.3)	6(24.0)	0.798

Data were expressed as mean ± SD, median with 25th and 75th percentile, n (%).

ACEI/ARB, angiotensin-converting enzyme inhibitors/angiotensin receptor blocker; ALT, alanine aminotransferase; ALP, alkaline phosphatase; ApoA1, apolipoprotein A1; ApoB, apolipoprotein B; AST, aspartate aminotransferase; BMI, body mass index; BUN, blood urea nitrogen; CCB, calcium channel blocker; Cr, creatinine; ESR, erythrocyte sedimentation rate; FFA, free fatty acids; FH, family history; GGT, glutamyl transferase; HbA1C, hemoglobin A1C; HDL-C, high-density lipoprotein cholesterol; LDL-C, low-density lipoprotein cholesterol; Lp(a), lipoprotein (a); OADs, oral antidiabetes drugs; SCAD: stable coronary artery disease;SD: standard deviation; T2DM:type 2 diabetes mellitus;TC, total cholesterol; TG, triglyceride; UA, uric acid.

p < 0.05 suggests significant difference. The meaning of the bold values were only for indicating the corresponding indicators have significant difference (p < 0.05).

### Association of HDL-C subfractions with CVEs in T2DM patients with SCAD

A logistic regression analysis was performed to further assess the association of HDL subfractions with CVEs in T2DM patients with SCAD (**Table 4**). In the present study, adjusted logistic regression analysis indicated that intermediate HDL-C concentrations and mixed HDL subfraction concentrations were positively related to the existence of CVEs in T2DM patients with SCAD [adjusted OR (95% CI): 1.154 (1.056–1.262), p=0.002; adjusted OR (95% CI): 1.095 (1.029–1.166), p=0.004; respectively].

**Table 4:** Associations of HDL subfractions with CVEs in T2DM and SCAD patients.

Variable	Unadjusted logistics regression analysis				Adjusted logistics regression analysis	
	OR	95% CI	p-value	OR	95% CI	p-value
HDL-C						
Large HDL-C(mg/dL)	1.007	0.945-1.073	0.835			
Intermediate HDL-C(mg/dL)	1.134	1.051-1.223	**0.001**	1.154	1.056-1.262	**0.002**
Small HDL-C(mg/dL)	1.140	1.009-1.228	**0.035**			
Mixed HDL (mg/dL)	1.091	1.031-1.154	**0.002**	1.095	1.029-1.166	**0.004**
Large HDL-C (%)	0.966	0.922-1.011	0.139			
Intermediate HDL-C (%)	0.980	0.894-1.075	0.674			
Small HDL-C (%)	1.008	0.959-1.060	0.745			
Mixed HDL (%)	1.012	0.970-1.056	0.579			

The adjusting known confounders were including age, gender, hypertension, family history of CAD, current smoking, BMI, drinking.

BMI, body mass index; CAD: coronary artery disease; CI, confidence intervals; HDL-C, high-density lipoprotein cholesterol; OR, odds ratio; SCAD, stable coronary artery disease; T2DM, type 2 diabetes mellitus.

p < 0.05 suggests significant difference. The meaning of the bold values were only for indicating the corresponding indicators have significant difference (p < 0.05).

### Relationship between HDL-C subfractions and outcomes of T2DM patients with SCAD

**Figure 2:**
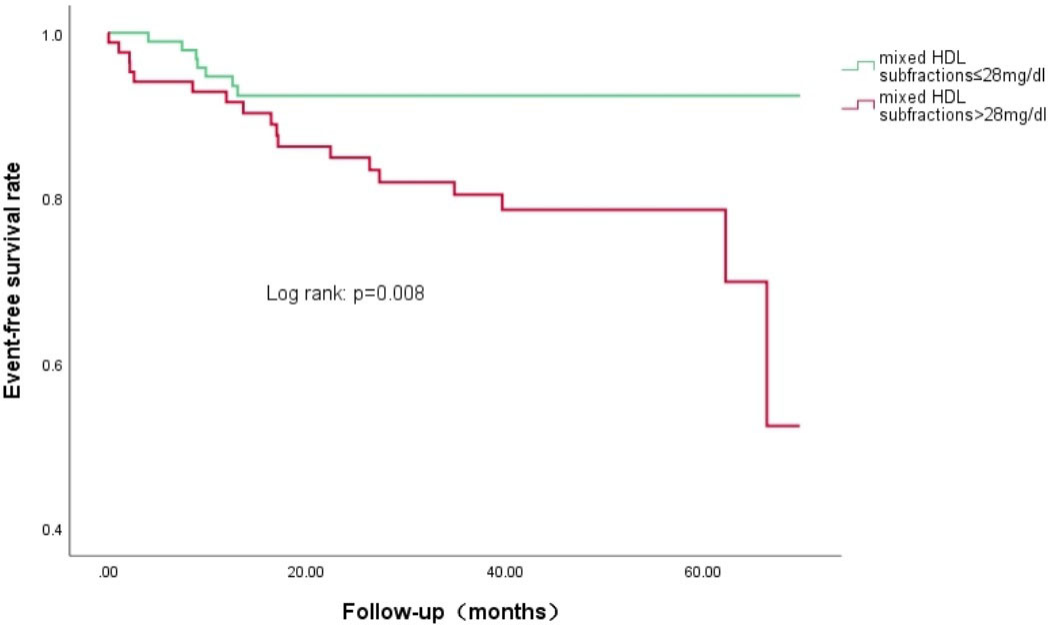
Kaplan-Meier curves of the cumulative event-free survival analyses according to mixed HDL subfractions levels in T2DM and SCAD patients at baseline.

As shown in **Table 5**, univariate Cox regression and multivariate Cox regression analyses revealed that the association of CVEs with intermediate HDL-C concentrations and mixed HDL subfraction concentrations remained significant after adjusting in three models [adjusted HR (95% CI): 1.097 (1.042–1.155); p<0.001; adjusted HR (95% CI): 1.061 (1.024–1.100); p<0.001; respectively]. Kaplan–Meier curve analysis of the probability of CVEs event-free survival during the follow-up period according to the low mixed HDL subfractions (≤ 28 mg/dL) and high mixed HDL subfractions (> 28 mg/dL) indicated that patients with higher levels of mixed HDL subfractions had lower event-free survival rates (p=0.008) (**Figure 2**).

**Table 5:** Cox regression models of HDL subfractions as predictors for CVEs in T2DM and SCAD patients.

Variable	Unadjusted Model			Model 1			Model 2			Model 3		
	HR	95%CI	p-value	HR	95%CI	p-value	HR	95%CI	p-value	HR	95%CI	p-value
HDL-C												
**Large HDL-C(mg/dL)**	1.003	0.951-1.059	0.898									
**Intermediate HDL-C (mg/dL)**	1.093	1.045-1.144	**<0.001**	1.090	1.044-1.137	**<0.001**	1.094	1.044-1.147	**<0.001**	1.097	1.042-1.155	**<0.001**
**Small HDL-C(mg/dL)**	1.122	1.018-1.237	**0.020**	1.107	1.005-1.220	**0.040**	1.108	1.002-1.225	**0.045**	1.110	0.996-1.236	0.058
**Mixed HDL (mg/dL)**	1.060	1.028-1.093	**<0.001**	1.056	1.025-1.087	**<0.001**	1.058	1.024-1.092	**<0.001**	1.061	1.024-1.100	**<0.001**
**Large HDL (%)**	0.972	0.935-1.010	0.106									
**Intermediate HDL-C (%)**	0.991	0.906-1.085	0.894									
**Small HDL-C (%)**	1.007	0.956-1.050	0.757									
**Mixed HDL (%)**	1.011	0.975-1.049	0.548									

Model 1 adjusted for age, gender; Model 2 adjusted for age, gender, hypertension, family history of CAD, current smoking; Model 3 adjusted for age, gender, hypertension, family history of CAD, current smoking, BMI, drinking.

BMI, body mass index; CAD, coronary artery disease; CI, confidence intervals; CVEs, cardiovascular events; HDL-C, high-density lipoprotein cholesterol; HR, hazard ratio; SCAD, stable coronary artery disease; T2DM, type 2 diabetes mellitus.

p < 0.05 suggests significant difference. The meaning of the bold values were only for indicating the corresponding indicators have significant difference (p < 0.05).

## Discussion

Many studies have investigated the changes in HDL-C subfractions or particles resulting from diabetes. We previously reported that the concentration of large HDL-C subfractions in SCAD patients is lower compared to healthy individuals but that the concentration of small HDL-C subfractions is higher in SCAD patients compared to healthy individuals (10). In the present study, we found that the concentration of large HDL-C subfractions was decreased and that the concentration of small HDL-C subfractions was increased in T2DM patients with SCAD compared to patients with SCAD alone. Moreover, the present data indicated that the concentrations of large HDL-C subfractions and small HDL-C subfractions were negatively and positively correlated, respectively, with the coexistence of T2DM and SCAD even after adjusting for traditional cardiovascular risk factors. More importantly, the present study is the first to report that the mixed HDL subfraction concentration predicts the risk of CVEs in T2DM patients with SCAD even after adjusting for traditional cardiovascular risk factors. Thus, the present findings suggested that measurement of HDL-C subfractions may be a novel useful tool for predicting clinical outcomes for T2DM patients with SCAD.

Data from numerous epidemiological and clinical studies have indicated that the mortality of SCAD is much higher in patients with T2DM, and the potential risk has been reported to be three times higher than that in patients without T2DM (11–13). The characteristics of dyslipidemia in T2DM patients consist of elevated concentrations of TG and low levels of HDL-C, which are significantly associated with the incidence of ASCVD (14). Previous study has also indicated that HDL has antiatherogenic and cardioprotective functions (15), while the role of HDL particles or subfractions in SCAD patients or T2DM patients with SCAD is not completely understood. HDL particles vary in size and function, which may be strongly associated with incident T2DM (16). Interestingly, we have previously reported that there is a significant increase in small HDL-C subfraction concentrations and a decrease in large HDL subfraction percentages in Chinese Han T2DM patients (9, 17). A previous study performed in 51 healthy subjects and 98 patients with T2DM or coronary heart disease using nuclear magnetic resonance (NMR) spectroscopy revealed a shift of HDL particle composition with a loss of large and very large particles as well as a gain of small triglyceride-rich particles in T2DM patients (18). Another study has reported that the HDL-C subfraction in T2DM patients with SCAD consists mostly of small triglyceride-rich HDL particles with a loss of large and very large HDL particles (19). Furthermore, Syvänne et al. (20) found that HDL particles in T2DM patients with SCAD are small in size and have lower free cholesterol content. Similarly, the present study found that the concentration and percentage of large HDL-C subfractions were significantly lower in T2DM patients with SCAD compared to SCAD patients, whereas the concentration and percentage of small HDL-C subfractions were higher in T2DM patients with SCAD compared to SCAD patients. Finally, the concentration and percentage of HDL-C large subfractions were negatively correlated with the coexistence of T2DM and SCAD, while the concentration and percentage of small HDL-C was positively correlated with the coexistence of T2DM and SCAD.

Although previous studies have shown a significant association between HDL lipoprotein subfractions and the coexistence of T2DM and SCAD, the prognostic value of different HDL-C subfractions in the outcome of T2DM patients with SCAD has been investigated less. The LUdwigshafen RIsk and Cardiovascular Health (LURIC) study reported that high concentrations of HDL-C particles are inversely related to cardiovascular mortality in SCAD patients, which is primarily driven by the smallest HDL-C particles (21). The Insulin Resistance Atherosclerosis Study (IRAS) showed a relationship between small HDL and incident diabetes in 830 individuals who had no diabetes at baseline and subsequently developed diabetes after a mean follow-up of 5.2 years (22). In a prospective study of 26,836 initially healthy women with 13 years follow-up for incident T2DM, the data showed that the small HDL particles were positively associated with diabetes (23). Moreover, higher levels of small HDL particles at baseline were associated with a higher risk of future T2DM during a median 7.3-year follow-up among 4828 subjects of the Prevention of Renal and Vascular End-Stage Disease (PREVEND) study without T2DM at baseline (24). The present study focused on the association of HDL-C subfraction concentrations with CVEs in SCAD patients with or without T2DM, and our data indicated that the concentrations of the mixed HDL subfractions were positively correlated with CVEs after adjustment for established cardiometabolic factors, which suggested that high levels of mixed HDL subfractions indicate higher risk of CVEs. These findings provide novel information regarding the relation of HDL-C subfraction and prognosis in T2DM patients with SCAD.

Recent studies reported that the changes of HDL particles or subfractions can predict the severity and outcomes of T2DM patients with or without SCAD, but the exact mechanisms underlying the HDL changes are not fully understood. It has been demonstrated that HDL exerts various antiatherogenic properties, including enhancing reverse cholesterol transport, antioxidative capacities and anti-inflammatory capacities in physiological conditions (25, 26). HDL may become dysfunctional in the pathological state which consists of a change in the composition of lipids and proteins in HDL, such as paraoxonase-1 and lipoprotein-associated phospholipase 2 (27–29). The size of HDLs in T2DM patients is altered with a loss of large and very large HDL2 (subclass of large and more buoyant HDL) as well as a shift toward small HDL3 (subclass of small and dense HDL) (30). This change may be a potential factor contributing to the proinflammatory properties of these particles in T2DM patients. It has been suggested that the predominant small HDLs are primary carriers of ceramides, which are recognized as potent activators of the NF-κB transcription factor involved in inflammation (31). The proteins in HDLs are significantly modified in T2DM patients with increased levels of serum amyloid A, fibrinogen, ApoC2 and ApoC3 levels as well as reduced levels of ApoA1, ApoA2, ApoE, ApoM and PON1 (32, 33). A previous study has shown that the function of large HDL2 protecting against LDL oxidation in individuals with T2DM is decreased compared to that of healthy controls, which is associated with decreased free cholesterol and increased TGs (34). A previous study using a small cohort of patients in whom insulin sensitivity was decreased has suggested another potential mechanism, in which insulin resistance may drive HDL subclass distribution towards smaller particles in T2DM patients (8). In a recent study of 8365 individuals aged 52 ± 13 years who have not taken lipid-lowering drugs, HDL2-C was reported to be inversely associated with exacerbation of insulin resistance but that HDL3-C showed the opposite results. Furthermore, after 5 years of follow-up, the HDL2-C level was negatively associated with a risk of T2DM incidence, clarifying the difference in prognostic significance of different HDL subclasses for exacerbation of insulin resistance and incidence of T2DM in the general population (35). Further investigation is needed to better understand the pathological mechanisms of HDL dysfunction, especially in T2DM patients with SCAD, in which there is an interaction between abnormal glucose metabolism and dyslipidemia.

Despite providing more information concerning changes of HDL subfractions in T2DM and SCAD, there were several limitations in the present study. First, the data was obtained from a single center, and there was a small sample size, indicating that the present findings should be confirmed by large-scale multicenter study in the future. Second, the short duration of follow-up, which was related to the small number of patients with CVEs, was another limitation. Third, because there were different measurements of lipoprotein subfractions, the present findings need to be confirmed using different methods. Additionally, the present study did not contain a group of T2DM patients without SCAD as a negative control, and there were no data for the plasma insulin and C-peptide levels as well as the mean duration of diabetes to evaluate the severity of T2DM. Finally, we did not assess the impact of lipoprotein subfraction on microvascular complications in T2DM patients with SCAD, suggesting that further study may be needed in the future.

## Conclusion

T2DM patients with SCAD have higher levels of mixed HDL subfractions, resulting in a higher risk of CVEs. Thus, an elevated concentration of the mixed HDL subfraction may be a novel prognostic marker for T2DM patients with SCAD.

## Data availability statement

The original contributions presented in the study are included in the article. Further inquiries can be directed to the corresponding authors.

## Ethics statement

The studies involving human participants were reviewed and approved by Ethics Committee of Fu Wai Hospital and Cardiovascular Institute, Beijing, China. The patients/participants provided their written informed consent to participate in this study. Written informed consent was obtained from the individual(s) for the publication of any potentially identifiable images or data included in this article.

## Author contributions

WZ analyzed the data and wrote the manuscript. R-XX and J-JL designed the study, interpreted the data, and contributed to critically revising the manuscript. J-LJ and H-WZ contributed to analyzing the data. YXZ, QD and JS contributed to data collection and procedure of laboratory examination. Y-LG and K-FD contributed to recruitment of patients. All authors contributed to the article and approved the submitted version.
