# Corrigendum: The value of HDL subfractions in predicting cardiovascular outcomes in untreated, diabetic patients with stable coronary artery disease: an age- and gender-matched case-control study

**DOI:** 10.3389/fendo.2023.1208564

**Published:** 2023-06-01

**Authors:** Wei Zhang, Jing-Lu Jin, Hui-Wen Zhang, Ya-Xin Zhu, Qian Dong, Jing Sun, Yuan-Lin Guo, Ke-Fei Dou, Rui-Xia Xu, Jian-Jun Li

**Affiliations:** State Key Laboratory of Cardiovascular Disease, Cardiometabolic Medicine Center, Fuwai Hospital, Chinese Academy of Medical Sciences & Peking Union Medical College, Beijing, China

**Keywords:** cardiovascular events, coronary artery disease, diabetes, high-density lipoprotein subfractions, predicting value

In the published article, several author names were written incorrectly in the author list. Instead of “Wei Zhang, Jinglu Jin, Huiwen Zhang, Yaxin Zhu, Qian Dong, Jing Sun, Yuanlin Guo, Kefei Dou, Ruixia Xu* and JianJun Li*”, the author list should have been “Wei Zhang, Jing-Lu Jin, Hui-Wen Zhang, Ya-Xin Zhu, Qian Dong, Jing Sun, Yuan-Lin Guo, Ke-Fei Dou, Rui-Xia Xu* and Jian-Jun Li*”.

In addition to this, there was an error in **Figure 1** as published. In the published version, some words are incorrectly shown in red. The corrected **Figure 1** and its caption appear below.

**Figure 1:**
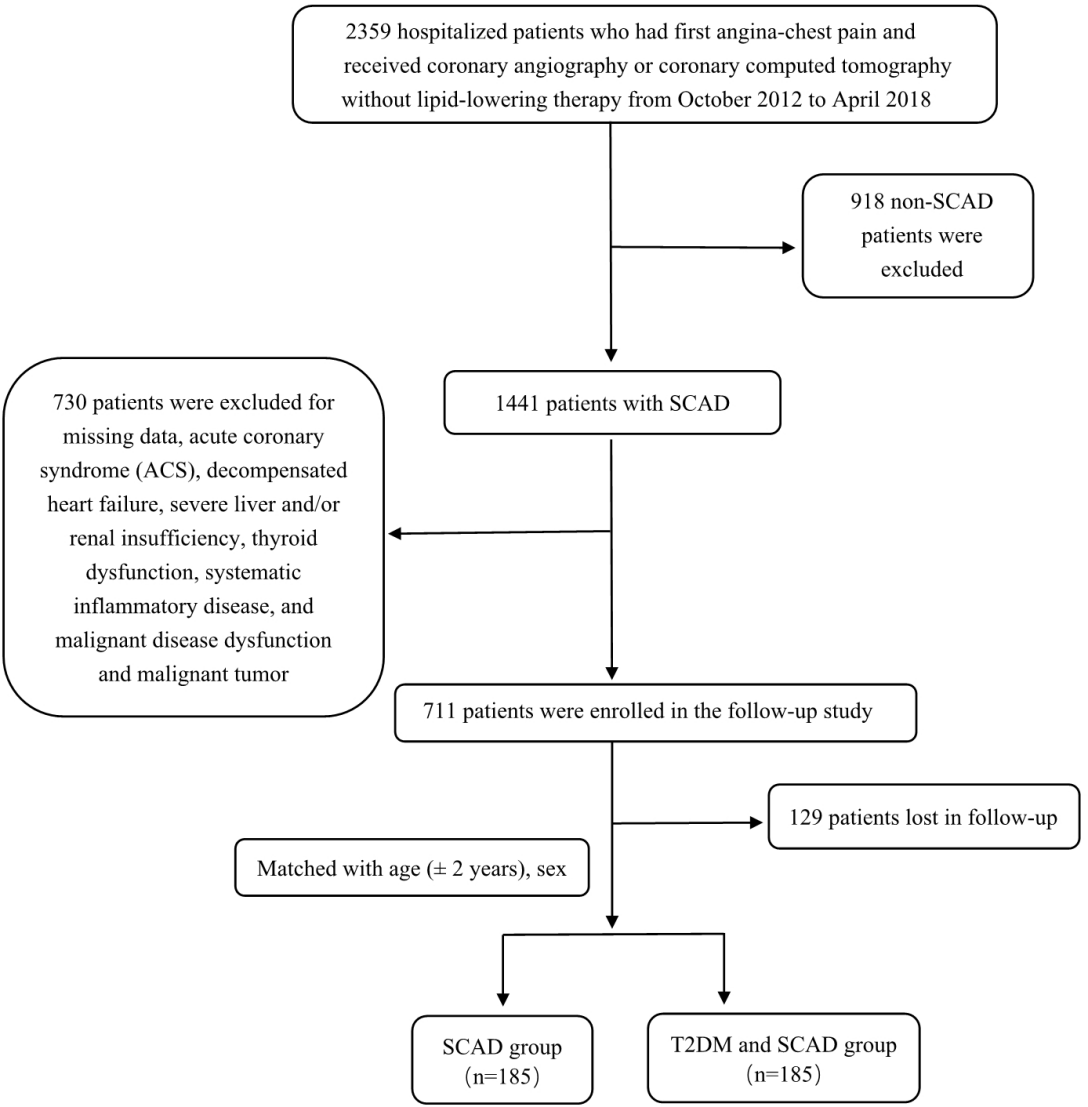
Flowcharts of patient selection and grouping.

Furthermore, in the published article there was an error in the author contributions section.

The author contributions statement was previously written as “WZ completed the project, analyzed the data, and wrote the manuscript. RX and JJ and HZ contributed to analyzing the data, and contributed to reviewing/editing the manuscript. JL and HZ contributed to analyzing the data. YZ, QD and JS contributed to data collection and procedure of laboratory examination. YG and KD contributed to recruitment of patients. All authors contributed to the article and approved the submitted version.”

The corrected author contributions statement is “WZ analyzed the data and wrote the manuscript. R-XX and J-JL designed the study, interpreted the data, and contributed to critically revising the manuscript. J-LJ and H-WZ contributed to analyzing the data. Y-XZ, QD and JS contributed to data collection and procedure of laboratory examination. Y-LG and K-FD contributed to recruitment of patients. All authors contributed to the article and approved the submitted version.”

Lastly, in the published article there was also an error in the Abstract, in the conclusions section. This sentence previously stated:

“Elevated concentration of the mixed HDL subfraction concentration predicts events in T2DM patients with SCAD.”

The corrected sentence appears below:

“Elevated concentration of the mixed HDL subfraction predicts events in T2DM patients with SCAD.”

The authors apologize for these errors and state that they do not change the scientific conclusions of the article in any way. The original article has been updated.

